# Development of an early prediction model for endometriosis risk: the simplified adolescent factors for endometriosis (SAFE) score

**DOI:** 10.1016/j.eclinm.2026.103806

**Published:** 2026-03-09

**Authors:** Gita D. Mishra, Mohammad Reza Baneshi, Sally Mortlock, Grant W. Montgomery, Jenny Doust, Annette J. Dobson, Jason Abbott

**Affiliations:** a Australian Women and Girls' Health Research Centre, School of Public Health, The University of Queensland, Brisbane, QLD 4006, Australia; b Institute for Molecular Bioscience, The University of Queensland, Brisbane, QLD 4072, Australia; c School of Clinical Medicine, Medicine and Health, Discipline of Women's Health, University of New South Wales Sydney, NSW 2033, Australia; d National Endometriosis Clinical and Scientific Trials Network, University of New South Wales Sydney, NSW 2033, Australia

**Keywords:** Endometriosis, Young women, Decision support aid, Primary care

## Abstract

**Background:**

Endometriosis affects 11% of women of reproductive age. Diagnosis is often delayed by 6–8 years due to nonspecific symptoms and lack of early detection tools. This study aimed to develop an early risk prediction model for endometriosis to support referral decisions in primary care.

**Methods:**

Data were from an endometriosis-focused sub-study of the Australian Longitudinal Study on Women's Health. Data were split into a training (75% of women born 1989–95, n = 4005), internal test (remaining 25%, n = 1335), and external test (women born 1973–78, n = 4077) samples. Stable risk factors were identified using a bootstrapping procedure with multivariable logistic regression. A logistic regression model based on these factors was used to develop the Simplified Adolescent Factors for Endometriosis (SAFE) score, calculated as a count of risk factors. Models were evaluated in all three samples.

**Findings:**

Six stable risk factors were identified; three factors related to severity of general pelvic pain, two with menstrual disorders, and one with a family history of endometriosis. For the final logistic regression model, the areas under the curves (AUCs) were 0.81, 0.78, and 0.72 for the training, internal, and external test samples, respectively. Using the SAFE score (values 0–6), the corresponding AUCs were 0.79 (95% CI: 0.77–0.82), 0.79 (95% CI: 0.75–0.83), and 0.71 (95% CI: 0.69–0.73). Using a cut-point of ≥2 for the SAFE score, the corresponding sensitivity was 59.6, 64.2, and 46.7, specificity was 83.4, 82.6, and 85.7, and negative predictive value was 94.6, 94.4, and 89.7.

**Interpretation:**

The SAFE score is an evidence-based tool to guide endometriosis referrals in primary care. It shows strong statistical performance and warrants clinical evaluation.

**Funding:**

The ALSWH study is funded by the Australian Government Department of Health, Disability, and Ageing. The GELLES study is supported by the Medical Research Future Fund (MRFF) (MRFF1199785). GDM and GWM are Australian National Health and Medical Research Council (NHMRC) Fellows (APP2009577; GNT1177194). SM is a National Endometriosis Clinical and Scientific Trials Network Fellow.

**Research in context**

**Evidence before this study**

To identify existing tools for early diagnosis of endometriosis, we conducted a structured literature search across PubMed, Scopus, and Embase to identify studies reporting clinical predictive factors for endometriosis based on patient-completed questionnaires, screening tools, or predictive models. The search was limited to English-language publications from 2003 to 2024. Titles were required to include the term *“endometriosis”* and at least one keyword related to diagnosis, prediction, or screening (e.g., *“diagnosis,” “prediction,” “screening,” “risk,” “predictive,” “diagnostic”*), and one keyword related to patient-reported variables or methodology (e.g., *“questionnaire,” “survey,” “symptom,” “assessment,” “model,” “algorithm,” “clinical”*). While some prediction models for endometriosis exist, ranging from complex models to those based on small samples, most have not been validated, implemented in clinical practice, or assessed in adolescent populations.

**Added value of this study**

The SAFE score is a simple, adolescent-focused risk prediction tool for endometriosis, developed using data from over 9000 women. Unlike previous models, SAFE is tailored for use in primary care, relies on easily reported symptoms and family history, and has strong statistical performance, offering a practical approach to reduce diagnostic delays, rather than confirming disease.

**Implications of all the available evidence**

The SAFE score offers a practical, evidence-based tool to support earlier identification and referral of young women at risk of endometriosis in primary care, reducing diagnostic delays. This study supports the need for further clinical evaluation and implementation research to inform practice guidelines and health policy.

## Introduction

Endometriosis is a debilitating condition affecting 6–11% of women of reproductive age.^1^^,^^2^ It is characterised by the growth of endometrial-like tissue outside the uterus, leading to dysmenorrhoea, dyspareunia, chronic pelvic pain, and bowel and urinary symptoms. It is associated with reduced quality of life, depression, infertility, and reduced workforce participation, leading to a substantial economic burden.^3^^,^^4^

On average, patients wait 6–8 years from symptom onset to diagnosis due to nonspecific symptoms, lack of awareness, misdiagnosis, and normalisation of menstrual pain.^5^, ^6^, ^7^, ^8^ This delay results in ongoing effects on women's physical health and quality of life, possible progression of disease,^9^ and disadvantages during fertility treatment.^10^^,^^11^ Research with women who have experienced endometriosis has identified reducing the diagnostic delay as a major priority.^12^^,^^13^

Determining which patients should receive initial treatment (such as with hormones) and who should be referred more urgently for further investigation is challenging. As a result, both health policy and clinical practice have increasingly focussed on the need to improve pathways for endometriosis diagnosis. Clinical guidelines on endometriosis cite multiple occasions when the authors had to rely on their experience and opinion for what constitutes good practice due to lack of evidence.^14^, ^15^, ^16^, ^17^

There have been numerous prediction models for screening or diagnosis of endometriosis.^18^^,^^19^ Many are complex, involving multiple risk factors, imaging findings, biomarkers, and clinical variables (e.g., Goldstein and Cohen).^20^ Others are based on small samples (e.g., Fauconnier, Drioueche).^21^ Most lack validation or evaluation in clinical practice. The aim of this study was to use robust methods to develop a decision aid appropriate for use in primary care to triage adolescent girls and young women for early diagnosis and treatment.

## Methods

### Study population

The Genetic Variants, Early Life Exposures and Longitudinal Endometriosis Symptoms (GELLES) Study is a sub-study of the Australian Longitudinal Study on Women's Health (ALSWH), a population-based study involving four age cohorts to examine determinants of health and health service use across the life course.^22^^,^^23^ Women were eligible for GELLES if they were participants in the ALSWH cohorts born in 1989–95 or 1973–78. Women were excluded if they had withdrawn from ALSWH, did not have a valid email address, had not consented to record linkage to administrative health records, were uncontactable or deceased. It was explained that to achieve the goals of GELLES, the research team needed women with and without endometriosis to participate in the study. Of the 17,010 women who participated in the 1989–95 cohort, 14,685 were eligible and 5340 (36.3%) responded to the survey in 2021–2022 (at age 26–33 years). Of the 14,247 women who participated in the 1973–78 cohort, 8751 were eligible and 4077 (46.5%) responded to the survey in 2023–2024 (at age 45–51 years). Participants were invited to complete the online GELLES survey, which included questions about in utero and early life factors, childhood exposures and social environment, family health, and detailed questions about their menstrual cycles and menstrual symptoms at various life stages. GELLES was approved by the Human Research Ethics Committees at the University of Newcastle (H-2021-0246) and the University of Queensland (2021/HE002488), and informed consent was obtained from participants. Ethical approval for data linkage was also obtained from the Human and Research Ethics Committees at the University of Newcastle (H-2011-0371) and The University of Queensland (Number: 2012000132).

### Outcome

Women in both cohorts were asked, “Has a doctor or other health care provider ever diagnosed you with endometriosis?” Those who reported a diagnosis were asked about the diagnostic method. The self-reported endometriosis diagnosis was categorised as surgically confirmed (i.e., diagnosed via laparoscopy or other surgical procedures), clinically suspected (i.e., diagnosis may have been based on MRI, ultrasound, or symptoms), or no endometriosis. If women did not respond to the question, the endometriosis status was determined using previous ALSWH survey responses and/or linked records of hospital admissions, investigations or procedures, or prescription of a restricted medication for endometriosis subsidised by Australia's universal health insurance system.^2^

Women with clinically suspected endometriosis (176 from the 1989–95 cohort and 144 from the 1973–78 cohort) may have had their diagnosis confirmed later (for example, if they were on a waiting list for further investigation or had been diagnosed via imaging). Therefore, in the primary analysis, women having endometriosis included those with both surgically confirmed and clinically suspected endometriosis.

### Risk factors

Risk factors were reported retrospectively for broad life-stage categories. Women in both cohorts were asked about their experience of various types of pain and symptoms before the age of 15, at ages 16–19, and 20–29. For the 1989–95 cohort, an additional category captured symptoms and after age 30, whereas for the 1973–78 cohort, additional categories captured symptoms at ages 30–39 and after 40. Potential risk factors for endometriosis focused on pelvic pain and its consequences for daily activities. Respondents rated the consequences on a scale from 0 to 10 and were categorised into three groups (0–3, 4–6, and 7–10) in order of increased severity. They also reported menstrual patterns, regularity of periods, menstrual flow, age at menarche, and if they had typically experienced period pain and the age when this began. Other potential risk factors included were preterm birth, exposure to smoking in utero or in early life, and the family history of endometriosis (see Supplementary Text S1).

### Statistical analysis

#### Step 1: handling of structural and non-structural missing data

Before being asked questions about menstrual characteristics (i.e., regularity of periods, menstrual flow, and cycle length), women were first asked “Did you have natural periods during this timeframe (i.e., periods that occurred when you were not on hormonal contraception)?”. A skip pattern was implemented such that women who selected ‘No’ or ‘used hormonal contraception for entire time frame’ did not proceed to answer menstrual characteristics. To deal with structural missing values resulting from the skip pattern, a new category ‘on contraception’ was defined for menstrual characteristics.

Non-structural missing data had a consistent pattern. For example, there were seven questions related to pelvic pain and its consequences for each life stage. Women who did not answer one of these questions almost always left the other questions unanswered as well. For each risk factor, a missing category was defined to deal with missing data.

#### Step 2: selection of the life stage with the highest predictive ability

**Fig. 1:**
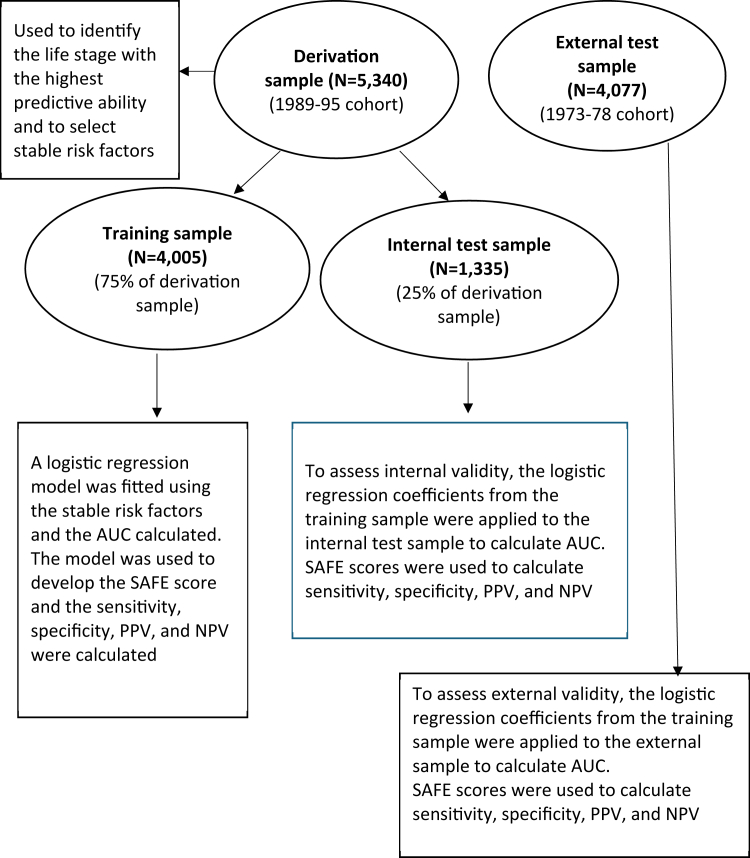
Development and validation of a model to predict endometriosis. AUC: area under curve; PPV: positive predictive value; NPV: negative predictive value; SAFE: Simplified Adolescent Factors for Endometriosis.

Fig. 1 illustrates the process of model development and validation using four sample datasets.

– The *derivation sample* - data from GELLES participants in the 1989–95 cohort were used to identify predictive risk factors.
– A *training sample* - a random sample of 75% of the derivation sample.
– An *internal test sample* - the remaining 25% of the derivation sample.
– An *external test sample* - GELLES participants in the 1973–78 cohort.

To ensure that the risk factors were experienced before the outcome (endometriosis diagnosis), only data collected for up to the age of 15, and 16–19 years were considered. Because symptoms across adjacent life stages were highly correlated, including both life-stage measures simultaneously could have introduced multicollinearity and unnecessary model complexity. We therefore used a filtering step to identify the adolescent life stage that provided the strongest predictive information. Within the derivation sample of the 1989–95 cohort, univariate logistic regression was used to estimate the crude association between each risk factor and endometriosis and the area under the curve (AUC) was calculated for each age range. For each risk factor, the measure from the adolescent life-stage category that yielded the highest AUC was selected. In addition, reports from ages up to 15 and 16–19 were combined to construct measures reflecting symptoms up to age 19, to examine whether a broader age band improved predictive performance.

#### Step 3: selection of stable risk factors

Stepwise variable selection methods, such as backward or forward selection, may result in unstable prediction models.^24^ Therefore, to assess the stability of risk factors to the variations in the sample, 100 bootstrap samples were drawn from the derivation sample.^25^ A multivariable logistic regression model, including all candidate risk factors, was fitted to each of the bootstrap samples. Risk factors that had a p-value < 0.05 in at least 50% of bootstrap samples were considered stable.^26^

#### Step 4: assessment of internal and external validity

Next, using the stable risk factors, a logistic regression model was developed using the training sample (75% random sample of the derivation data) and the odds ratios (OR) and 95% confidence intervals (CI) were estimated. The coefficients from this model were then applied to the training, internal test sample and the external sample. The AUC was calculated from the predicted probabilities to evaluate the performance of the coefficient-based model.

Moreover, to develop the simplified adolescence risk factors for endometriosis (SAFE) score, each of the six stable risk factors was assigned a score of one and the SAFE score was defined as the sum (range 0–6). For heavy menstrual flow, a point was assigned only when the symptom was explicitly reported as present. This variable had three categories (‘no’, ‘yes’, and ‘on contraception’); only women who selected ‘yes’ received one point, whereas ‘no’ and ‘on contraception’ received no points because the latter reflected hormonally suppressed bleeding patterns rather than symptom presence. The performance statistics for SAFE were assessed with a cut-off at 1 point, 2 points, and 3 points using the AUC, sensitivity, specificity, negative predictive value (NPV), and positive predictive value (PPV). To identify the optimal threshold for distinguishing low and high-risk women, we selected the cut-point that maximised the Youden Index, a measure that summarises the balance between sensitivity and specificity.

#### Step 5: sensitivity analysis

A series of sensitivity analyses were conducted to examine the robustness of the model predictions.

1. Firstly, the selection process for stable risk factors was applied to the derivation sample after excluding women with missing data.
2. Second, the process of variable screening, bootstrap stability selection, and model fitting was applied to the external sample (1973–78 cohort) to identify the set of stable risk factors in the older cohort. Based on these stable predictors, we constructed an alternative risk score by summing the number of risk factors present. This score was then calculated for women in the 1989–95 cohort, and its discriminative performance was evaluated by estimating the AUC.
3. Third, to address the potential impact of recall bias for risk factors reported for ages 16–19, the development of the training sample model was repeated but using the most recent reported values of risk factors available before the age of endometriosis diagnosis. For women without endometriosis, risk factors were taken from the most recent life stage. This sensitivity analysis approximated a model in which, for each participant, risk factors measured before diagnosis were used as baseline predictors instead of risk factors reported during the ages of 16–19.
4. Lastly, to examine whether diagnostic misclassification affected predictor selection or model performance, a model was developed again with the training sample, but women with clinically suspected endometriosis were considered as not having endometriosis. This analysis limited the case definition to surgically confirmed endometriosis and allowed assessing whether predictor stability and model performance were robust to a more stringent diagnostic definition. This revised model was then tested and evaluated using the internal and external test samples.

All analyses were conducted using the following R packages: boot (to draw bootstrap samples), pROC (to estimate AUC and other performance statistics), and ggplot2 (for visualisation).

### Role of the funding source

The funders had no role in the study design, data collection, data analysis, data interpretation, or writing of the manuscript.

## Results

There were differences between the two cohorts in baseline sociodemographic and health-related characteristics (Table 1). Women in the 1989–95 cohort were more likely to live in major cities and to report financial stress, and obesity was more common, whereas current smoking and drinking at a risky level of alcohol were less common.

**Table 1:** Baseline characteristics of women who participated in the GELLES survey.

Variable	Category	1989–95 cohort (N = 5340)	1973–78 cohort (N = 4077)
Marital status	Single	1133 (21.2%)	809 (19.8%)
	S/D/W	10 (0.2%)	19 (0.5%)
	Married	4159 (77.9%)	3234 (79.3%)
	Missing	38 (0.7%)	15 (0.4%)
Area of residence	Major cities	3951 (74.0%)	2174 (53.3%)
	Inner regional	916 (17.2%)	1224 (30.0%)
	Outer regional	357 (6.7%)	544 (13.3%)
	Remote/very remote	54 (1.0%)	132 (3.2%)
	Overseas	58 (1.1%)	
	Missing	4 (0.1%)	3 (0.7%)
Ability to manage on income	Impossible/always difficult	1157 (21.7%)	676 (16.6%)
	Sometimes difficult	1842 (34.5%)	1336 (32.8%)
	Not bad	1632 (30.6%)	1457 (35.7%)
	Easy	671 (12.6%)	599 (14.7%)
	Missing	38 (0.7%)	9 (0.2%)
Body mass index	Underweight	397 (7.4%)	348 (8.5%)
	Normal	3229 (60.5%)	2589 (63.5%)
	Overweight	997 (18.7%)	559 (13.7%)
	Obesity	619 (11.6%)	225 (5.5%)
	Missing	98 (1.8%)	356 (8.7%)
Smoking status	Never	3619 (67.8%)	2210 (54.2%)
	Ex	937 (17.6%)	588 (14.4%)
	Current	750 (14.0%)	1124 (27.6%)
	Missing	34 (0.6%)	155 (3.8%)
Alcohol consumption	Low	3056 (57.2%)	2206 (54.1%)
	None	367 (6.9%)	295 (7.2%)
	Rare	1730 (32.4%)	1323 (32.5%)
	Risky/very risky	153 (2.9%)	211 (5.2%)
	Missing	34 (0.6%)	42 (1.0%)

S/D/W: separated, divorced, widowed.

In the primary analysis that included women with clinically suspected endometriosis as having endometriosis, the proportion of women with endometriosis was 11.0% (585 out of 5340) in the 1989–95 cohort (derivation sample) and 16.1% (658 out of 4077) in the 1973–78 cohort (external sample). There were differences in the age at diagnosis of endometriosis: the first quartile, median and third quartile for the 1989–95 cohort were 20, 24.07, and 27.54 years compared with 23, 32, and 38 years for the 1973–78 cohort. Women in the 1973–78 cohort were, on average, 8.1 years (95% CI: 6.65, 9.54) older at diagnosis than women in the 1989–95 cohort.

In the derivation sample, pelvic pain and menstrual symptoms reported for ages 16–19 years had higher predictive value (measured by AUC) than those for ages reported to up to 15 years (Supplementary Table S1). Combining reports from ages up to 15 with those from 16 to 19 years (i.e., reflecting symptoms up to age 19) only changed the third decimal point of AUC, indicating no meaningful improvement in predictive performance. Therefore, the age range 16–19 (or up to 19 for period pain due to question wording) was used for subsequent analyses.

Prevalence of endometriosis, univariate (crude) estimates of ORs, 95% CIs and AUC values for risk factors experienced by women in the derivation sample at ages 16–19 years are shown in Table 2 - all except having been born preterm were statistically significant.

**Table 2:** Univariate diagnostic performance of each risk factor associated with endometriosis in the derivation sample (1989–95 cohort; N = 5340).

Risk factor	Proportion with endometriosis	Crude OR (95% CI)	Area under curve (AUC)
Risk factors related to pelvic pain and its consequences			
Between the ages of 16 and 19, how often did you have pelvic pain? (do not count pain related to period, intercourse, pregnancy or childbirth, surgery, injury, food poisoning, or stomach flu)			
Never/rarely/sometimes (n = 4546)	8.4%	1.00	0.62
Often/very often (n = 391)	41.9%	7.77 (6.21, 9.72)	
Missing (n = 403)	7.5%		
Between the ages of 16 and 19, how long did an episode of pelvic pain usually last?			
<1 h (n = 4367)	8.1%	1.00	
≥1 h (n = 561)	33.7%	5.62 (4.59, 6.89)	0.62
Missing (n = 412)	7.7%		
Between the ages of 16 and 19, did you seek treatment for this pain?			
No (n = 4428)	7.7%	1.00	
Yes (n = 498)	40.2%	7.77 (6.32, 9.56)	0.64
Missing (n = 414)	7.8%		
When you had this pelvic pain between the ages of 16 and 19, how difficult did the pain make it for you to participate in daily work or school activities?			
0–3 (n = 4065)	6.9%	1.00	
4–6 (n = 476)	22.7%	3.76 (2.95, 4.79)	
7–10 (n = 390)	39.2%	8.27 (6.55, 10.43)	0.67
Missing (n = 409)	7.3%		
When you had this pelvic pain between the ages of 16 and 19, how difficult did the pain make it for you to participate in daily social or recreational activities?			
0–3 (n = 4097)	7.0%	1.00	
4–6 (n = 455)	23.8%	3.97 (3.11, 5.07)	
7–10 (n = 388)	38.7%	8.00 (6.34, 10.10)	0.66
Missing (n = 410)	7.7%		
Thinking about your pelvic pain between the ages of 16 and 19, please rate how severe your pelvic pain was			
0–3 (n = 3833)	6.6%	1.00	
4–6 (n = 522)	16.9%	2.71 (2.10, 3.51)	
7–10 (n = 574)	35.2%	7.27 (5.90, 8.95)	0.67
Missing (n = 411)	7.7%		
Did you ever take painkillers for pelvic pain?			
No (n = 3782)	6.5%	1.00	
Yes (n = 1142)	25.9%	4.73 (3.96, 5.66)	0.66
Missing (n = 416)	7.8%		
Menstrual related risk factors			
Pelvic pain during period (Painful periods) up to age 19			
No (n = 3512)	4.6%	1.00	
Yes (n = 1362)	27.0%	6.42 (5.35, 7.70)	0.71
Missing (n = 466)	8.7%		
Period irregularity between the ages of 16 and 19			
Regular (n = 2087)	6.5%	1.00	
Irregular (n = 674)	12.0%	1.79 (1.35, 2.37)	
No pattern (n = 185)	17.8%	2.84 (1.89, 4.28)	
On contraception (n = 2126)	14.3%	2.19 (1.79, 2.67)	0.60
Missing (n = 268)	5.0%		
Cycle length between the ages of 16 and 19			
≥24 days (n = 2447)	7.1%	1.00	
<24 days (n = 146)	17.1%	2.52 (1.60, 3.97)	
Too irregular (n = 347)	14.4%	2.06 (1.48, 2.86)	
On contraception (2126)	14.3%	2.04 (1.69, 2.46)	0.60
Missing (n = 274)	5.1%		
Menstrual flow between the ages of 16 and 19			
Spotting/light/moderate (n = 2531)	6.9%	1.00	
Heavy (n = 399)	19.3%	3.06 (2.30, 4.08)	
On contraception (2126)	14.3%	2.14 (1.77, 2.58)	0.61
Missing (n = 284)	5.3%		
Age at menarche			
>11 years (n = 4345)	10.5%	1.00	
≤11 years (n = 958)	13.0%	1.28 (1.04, 1.58)	0.52
Missing (n = 37)	0.7%		
Risk factors related to exposure to smoking in utero or in early life			
Mother smoking during pregnancy			
No (including don't know) (n = 3948)	10.6%	1.00	
Yes (n = 593)	15.2%	1.54 (1.21, 1.96)	0.52
Missing (n = 424)	7.9%		
Mother smoking during childhood			
No (n = 4161)	10.5%	1.00	
Yes (n = 703)	15.1%	1.54 (1.23, 1.93)	0.53
Missing (n = 476)	8.9%		
Father smoking during childhood			
No (n = 4199)	10.5%	1.00	
Yes (n = 665)	15.0%	1.53 (1.21, 1.93)	0.53
Missing (n = 476)	8.9%		
Other risk factors (family history of endometriosis and preterm birth)			
History of endometriosis in other family members (i.e., mother, sister, or grandmother/aunts/cousins on either maternal or paternal sides)			
No (including don't know/not applicable/missing) (n = 4296)	7.4%	1.00	
Yes (n = 1044)	25.5%	4.26 (3.56, 5.10)	0.65
Having been born preterm			
No (n = 4358)	11.1%	1.00	
Yes (n = 467)	10.9%	0.99 (0.73, 1.35)	0.50
Missing (n = 515)	9.6%		

The bootstrap process for identifying stable risk factors yielded six: three related to general pelvic pain symptoms between the ages of 16 and 19 (experienced pelvic pain often/very often, sought treatment for pelvic pain, and took painkillers for pelvic pain), two related to menstrual disorders (experienced heavy menstrual flow between the ages of 16 and 19 and experienced painful periods by age 19) and the final stable risk factor was having a family history of endometriosis (including mother, sister, grandmother, aunts, or cousins on either maternal or paternal sides). The bootstrap confidence intervals for these six stable predictors did not include the null value of 1, whereas the intervals for other predictors did, and therefore those predictors were not considered further.

These six risk factors identified in the derivation sample were also associated with endometriosis in the training sample (Table 3).

**Table 3:** Association between stable risk factors and endometriosis in the training sample (75% of the derivation sample of data from the 1989–95 cohort; N = 4005).

Risk factor	OR (95% CI)
How often did you have pelvic pain? (do not count pain related to period, intercourse, pregnancy or childbirth, surgery, injury, food poisoning, or stomach flu)	
Never/rare/sometimes	1.00
Often/very often	1.76 (1.21, 2.57)
Did you seek treatment for this pain?	
No	1.00
Yes	2.10 (1.44, 3.07)
Did you ever take painkillers for this pain?	
No	1.00
Yes	1.66 (1.22, 3.07)
Menstrual flow	
Spotting/light/moderate	1.00
Heavy	1.25 (0.97, 1.70)
On contraception	1.59 (1.25, 2.03)
Painful periods up to the age of 19	
No	1.00
Yes	4.16 (3.29, 5.27)
History of endometriosis in other family members	
No (including don't know)	1.00
Yes	3.29 (2.60, 4.16)

Stable risk factors were defined as those that had a p-value <0.05 in more than 50% of bootstrap samples.

The distributions of the six risk factors were similar for the training and internal test samples but there were differences in the external test sample (Table 4). For example, the proportion of women who reported experiencing heavy menstrual flow at age 16–19 years was 7.2% in the training sample and 13.0% in the external test sample. The proportion of women in the training sample who sought treatment for pelvic pain was higher at 9.1% compared with 5.9% in the external test sample. The distributions of all risk factors in both cohorts are provided in Supplementary Tables S2–S5.

**Table 4:** Prevalence of the six stable risk factors with 95% confidence intervals in the training sample (random sample of 75% of data from the 1989–95 cohort), internal test sample (the remaining 25% of data from the 1989–95 cohort), and external test sample (data from the 1973–78 cohort).

Risk factor	Training sample (N = 4005)	Internal test sample (N = 1335)	External test sample (N = 4077)
Between ages 16 and 19, how often did you have pelvic pain? (do not count pain related to period, intercourse, pregnancy or childbirth, surgery, injury, food poisoning, or stomach flu)			
Never/rare/sometimes	85.0% (83.9%, 86.1%)	85.4% (83.5%, 87.3%)	87.3% (86.3%, 88.4%)
Often/very often	7.3% (6.5%, 8.1%)	7.3% (5.9%, 8.7%)	5.9% (5.2%, 6.7%)
Between ages 16 and 19, did you seek treatment for this pain?			
Yes	9.1% (8.2%, 10.0%)	10.0% (8.4%, 11.6%)	5.9% (5.2%, 6.6%)
Between ages 16 and 19, did you ever take painkillers for this pain?			
Yes	21.2% (19.9%, 22.5%)	21.9% (19.7%, 24.2%)	15.2% (14.1%, 16.3%)
Menstrual flow between ages 16 and 19			
Heavy	7.2% (6.4%, 8.0%)	8.2% (6.8%, 9.7%)	13.0% (11.9%, 14.0%)
On contraception	39.7% (38.2%, 41.2%)	40.2% (37.6%, 42.9%)	23.0% (21.7%, 24.3%)
Painful period up to the age of 19			
Yes	25.0% (23.6%, 26.3%)	27.1% (24.7%, 29.5%)	21.9% (20.6%, 23.1%)
History of endometriosis in other family members			
Yes	19.2% (18.0%, 20.4%)	20.7% (18.5%, 22.8%)	17.5% (16.3%, 18.7%)

**Fig. 2:**
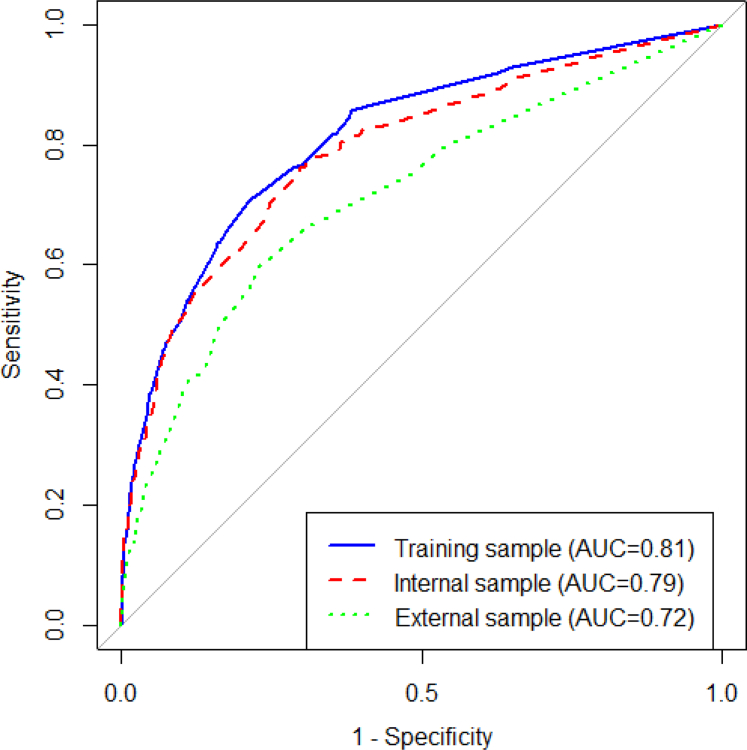
Performance of the model developed using the training sample to predict endometriosis in the training (75% of the derivation sample of the 1989–95 cohort), internal test (25% of the derivation sample), and external test samples (the 1973–78 cohort).

Despite these differences in symptom prevalence, the multivariable logistic regression model developed for the training sample performed well when its coefficients were applied to each data set (Fig. 2). In the training sample, the coefficient-based model resulted in an AUC of 0.81 (95% CI: 0.79, 0.84). It also showed good agreement between observed and predicted probabilities, indicating good model fit (Hosmer–Lemeshow p-value for calibration test = 0.56) (see Supplementary Text S2 and Supplementary Figure S1). When the same coefficients were applied to the internal test, the AUC was similar at 0.79 (95% CI: 0.75, 0.83) but was lower for the external sample at 0.72 (95% CI: 0.70, 0.74) (Fig. 2). The estimated regression coefficients and details on calculating the probability of endometriosis are provided in Supplementary Text S3.

The SAFE score was calculated as the sum of the six selected risk factors with one point for each factor reported, but no point for the menstrual flow symptom was assigned for women who reported being on contraception for the entire period of 16–19 years of age. This produced an overall risk score ranging from 0 to 6. Each 1-point increase in the SAFE score represented an approximate doubling of the prevalence of endometriosis (odds ratios about 2 for each data set) (Table 5).

**Table 5:** Proportion of women who reported endometriosis and odds ratios per additional SAFE score across different samples.

Number of risk factors	Training sample (75% of 1989–95 cohort; N = 4005; P = 10.6%)		Internal test sample (25% of 1989–95 cohort; N = 1335; P = 11.9%)		External test sample (1973–78 cohort; N = 4077; P = 16.1%)	
	P^a^	OR (95% CI)	P	OR (95% CI)	P	OR (95% CI)
0	$\frac{53}{2046}=2.6\%$		$\frac{24}{664}=3.6\%$		$\frac{178}{2227}=8.0\%$	
1	$\frac{119}{1111}=10.7\%$	4.51 (3.23, 6.29)	$\frac{33}{365}=9.0\%$	2.65 (1.54, 4.56)	$\frac{173}{1056}=16.4\%$	2.26 (1.80, 2.82)
2	$\frac{82}{423}=19.4\%$	2.00 (1.47, 2.72)	$\frac{29}{135}=21.5\%$	2.75 (1.60, 4.75)	$\frac{146}{447}=32.7\%$	2.48 (1.92, 3.20)
3	$\frac{50}{199}=25.1\%$	1.40 (0.94, 2.08)	$\frac{29}{86}=33.7\%$	1.86 (1.02, 3.41)	$\frac{69}{183}=37.7\%$	1.25 (0.88, 1.79)
4–6	$\frac{122}{226}=54.0\%$	3.5 (2.31, 5.29)	$\frac{44}{85}=51.8\%$	2.11 (1.14, 3.91)	$\frac{92}{164}=56.1\%$	2.11 (1.37, 3.24)

Odds ratios show the risk of endometriosis for adjacent categories comparing women with 1 risk factor versus no risk factors, two risk factors versus one, and so on.

SAFE: Simplified Adolescent Factors for Endometriosis.

a P shows the proportion of women who reported endometriosis. For example, in the training sample, 53 out of 2046 women with a score of 0 had a report of endometriosis, which corresponded to a proportion of 2.6%.

The SAFE score resulted in slightly lower AUC than using exact parameter estimates, with 0.79 (95% CI: 0.77, 0.82), 0.79 (95% CI: 0.75, 0.83), and 0.71 (95% CI: 0.69, 0.73) in the training, internal, and external test samples respectively (Table 6). As the cut-off to classify women into low and high-risk groups increased from 1 to 2 to 3 points the sensitivity of the model using the training sample declined from 87.5% to 59.6% to 40.4% respectively, while the specificity increased from 55.7% to 83.4% to 92.9%. Similar patterns were evident in the internal test and the external test samples. The Youden Index (which combines sensitivity and specificity) suggested the choice of a cut-point of 2 across all samples. When a cut-off of 2 was applied, in the training, internal, and external samples the PPVs were 29.9%, 33.3%, and 38.7% respectively, and the NPVs were 94.6%, 94.2%, and 89.7%.

**Table 6:** Assessment of internal and external validity of the SAFE model^a^ using the sum of the number of risk factors when women with suspected endometriosis were treated as having endometriosis.

Statistics	Cut-off point	Training sample (75% of 1989–95 cohort; N = 4005)	Internal test sample (25% of 1989–95 cohort; N = 1335)	External test sample (1973–78 cohort; N = 4077)
Proportion of women with endometriosis		10.6%	11.9%	16.1%
Sensitivity (%)	1	87.5 (84.4, 90.7)	84.9 (79.3, 90.5)	72.9 (69.6, 76.3)
	2	59.6 (55.0, 64.3)	64.2 (56.7, 71.6)	46.7 (42.8, 50.5)
	3	40.4 (35.7, 45.0)	45.9 (38.2, 53.7)	24.5 (21.2, 27.8)
Specificity (%)	1	55.7 (54.1, 57.3)	54.4 (51.6, 57.3)	59.9 (58.3, 61.6)
	2	83.4 (82.2, 84.6)	82.6 (80.5, 84.8)	85.7 (84.6, 86.9)
	3	92.9 (92.1, 93.8)	91.7 (90.1, 93.2)	94.5 (93.8, 95.3)
PPV (%)	1	19.0 (17.3, 20.8)	20.1 (17.1, 23.2)	25.9 (23.9, 27.9)
	2	29.9 (26.9, 33.0)	33.3 (28.1, 38.6)	38.7 (35.3, 42.1)
	3	40.4 (35.8, 45.1)	42.7 (35.3, 50.1)	46.4 (41.2, 51.6)
NPV (%)	1	97.4 (96.7, 98.1)	96.4 (95.0, 97.8)	92.0 (90.9, 93.1)
	2	94.6 (93.8, 95.3)	94.4 (93.1, 95.9)	89.7 (88.2, 90.4)
	3	92.9 (92.1, 93.7)	92.6 (91.1, 94.1)	87.7 (85.6, 87.8)
Yuden index	1	43.2 (39.7, 46.8)	39.3 (33.1, 45.6)	32.9 (29.1, 36.7)
	2	43.0 (38.2, 47.8)	46.8 (39.0, 54.6)	32.5 (28.5, 36.5)
	3	33.2 (28.5, 38.0)	37.5 (29.6, 45.4)	19.0 (15.7, 22.4)
AUC		0.79 (0.77, 0.82)	0.79 (0.75, 0.83)	0.71 (0.69, 0.73)

Values in parentheses indicate 95% CI.

In the main analysis, women with suspected endometriosis were considered as having endometriosis and performance statistics were calculated.

SAFE: Simplified Adolescent Factors for Endometriosis.

a The model was developed using training data and its performance was assessed on training, internal test, and external samples.

### Sensitivity analysis

In the first sensitivity analyses, when women with missing data were excluded, the same six risk factors were selected as stable in the derivation sample.

In the second sensitivity analysis, when data from the external sample (1973–78 cohort) were analysed using bootstrapping, the same stable risk factors were mostly identified (Supplementary Table S6). The exception was that taking painkillers for pelvic pain during the ages 16–19 was not selected. Using the five stable predictors identified in the 1973–78 cohort, the corresponding sum score showed an AUC of 0.78 (95% CI: 0.76, 0.80) when applied to the 1989–95 cohort.

In the third sensitivity analysis, when the most recent values of the risk factors relative to age at endometriosis diagnosis were used in the derivation model, the same six risk factors were selected (Supplementary Table S7), indicating that predictor selection was robust to the potential recall bias.

Finally, in the fourth sensitivity analysis, when women with clinically suspected endometriosis were treated as not having endometriosis, thereby restricting the case group to those with a surgically confirmed diagnosis, the same six risk factors were selected as being stable in the derivation sample. This analysis resulted in a model with better performance statistics: the SAFE score had an AUC of 0.82 (95% CI: 0.79, 0.84) in the training sample, 0.81 (95% CI: 0.77, 0.85) in the internal test sample, and 0.73 (95% CI: 0.71, 0.76) in the external test sample (Supplementary Table S8). Moreover, regardless of the cut-off point, the NPV was always above 90.0%.

## Discussion

Based on survey data from a community cohort of Australian women we developed a prediction model for endometriosis risk among adolescents. After a multi-step process, we identified six stable risk factors: experiencing pelvic pain often/very often, seeking treatment for pelvic pain, taking painkillers for pelvic pain, heavy menstrual bleeding, a history of painful periods and a family history of endometriosis.

By simplifying the coefficients from multiple logistic regression models, we developed the SAFE score as the sum of one point for each of the six risk factors. Each point increase in the SAFE score represented a marked increase in the prevalence of endometriosis, such that among women with ≥4 points, over half had endometriosis. The NPV was over 90% (≥94% for ≥2 points, and >92% for ≥3 points) in the training and internal test samples. The slightly lower NPV values for the external test sample (89.7% for ≥2 points and 87.7% for ≥3 points) were to be expected, as these women (aged 45–51 years) may have been diagnosed with endometriosis on average 8.1 years later in life without experiencing or recalling symptoms in adolescence. This difference likely reflects increased awareness, earlier recognition of symptoms, and improved diagnostic pathways in more recent decades.

To our knowledge, this is the first study to develop and evaluate a model for the risk of endometriosis based on a sum of risk factors experienced at ages 16–19 years. The SAFE score can be readily used in primary care without the need for invasive testing and little additional consultation time. The score should be regarded as a referral aid rather than a diagnostic test. The goal is to reduce diagnostic delay rather than to confirm the disease.

The next step in our programme of work is clinical validation of the SAFE Score for use in clinical settings and across diverse populations. It also seeks to translate the validated SAFE Score into a clinical decision support tool to guide early referral and investigation in adolescents and young women. The benefits of reducing the time to a definitive diagnosis may need to be balanced against the potential risk of causing unnecessary anxiety by introducing the label of endometriosis to an adolescent girl. This highlights the importance of responsible communication and appropriate clinical counselling. We will work closely with end-users to ensure that the tool and its messaging are implemented in a supportive manner.

A Cochrane review of biomarkers for the diagnosis of endometriosis found that none met the pre-determined criteria of sensitivity ≥0.95 and specificity ≥0.50, to rule out the diagnosis if there is a negative test result, or sensitivity ≥0.50 and specificity ≥0.95 to rule in the diagnosis if there is a positive result.^27^ The SAFE score almost achieves this level for a positive test, but not for a negative test. Therefore, women initially assessed as being low risk using this score need to be followed up over time to ensure that the diagnosis is not missed. The SAFE score, whilst based on symptoms in adolescence, is not restricted to use with this age group as these factors can be recalled by women later in life as demonstrated in our external test sample.

Our findings are consistent with previous studies that have identified a history of general pelvic pain, taking painkillers for pelvic pain, painful periods (dysmenorrhoea), heavy menstrual bleeding, and having a family history of endometriosis as associated with endometriosis.^18^, ^19^, ^20^, ^21^ The closest parallel is a Norwegian study of 313 women that focussed on dysmenorrhoea during adolescence.^28^ That study identified absenteeism from school due to dysmenorrhoea, severe dysmenorrhoea, and use of painkillers due to dysmenorrhoea as risk factors for endometriosis, and produced a model with comparable performance, e.g., AUC = 0.85 when a family history of endometriosis was included. The association between menstrual characteristics experienced before the age of 19 and endometriosis have also been found in earlier small-scale studies. A study of 134 Russian girls (from menarche to age 17 years) found peritoneal endometriosis was associated with a family history of the disease, persistent dysmenorrhoea, decreased daily activity, and gastrointestinal symptoms.^29^ Similarly, a study of 513 Australian women reported significant associations between younger age at menarche, shorter cycle length and dysmenorrhoea in adolescence and risk of endometriosis.^30^ While early menarche was initially identified as significant in the present study, it did not survive the multi-stage selection process.

This study has several strengths, notably the large, population-based sample with longitudinal data on symptoms. The model underwent rigorous validation, and its performance was assessed in an independent cohort of Australian women.

A limitation is that the ALSWH cohorts overrepresent women with higher education levels and underrepresent minority groups (e.g., non-European backgrounds and people with disabilities).^22^^,^^23^ Moreover, across both cohorts, GELLES participants had more advantaged sociodemographic and health profiles at baseline: a higher proportion reported managing on their income easily, and smoking was less common than among other ALSWH women. The potential misclassification of participants with undiagnosed endometriosis may also introduce bias, although the sensitivity analysis, including women with clinically suspected (but not formally diagnosed) endometriosis had minimal impact on the performance of the model. Another limitation is participants' recall of symptoms from earlier ages and knowledge of their family history. However, recalled symptoms at ages 16–19 and 20–29 were highly correlated with menstrual symptoms reported in real-time in past ALSWH surveys, supporting the reliability of retrospective reporting. Additionally, some early premenarcheal exposures, whether not captured or difficult to assess retrospectively, may represent missing determinants of risk. Although this study included an external validation sample, both the derivation and external test samples were drawn from GELLES participants within the broader ALSWH. Therefore, the external validation represents performance across independent cohorts within the same national population-based study rather than in geographically distinct populations. Further validation in independent populations and diverse geographical settings is required to strengthen broader applicability.

Another consideration relates to the accuracy of the self-reported endometriosis outcome used in this study. We recently conducted a separate validation study using GELLES data, comparing self-reported endometriosis against longitudinal survey data combined with linked administrative records (hospital admissions, procedures, and medication records) as the reference standard.^31^ This study demonstrated good validity and reliability for self-reported endometriosis. Agreement was strongest for surgically confirmed diagnoses. Clinically suspected diagnoses showed lower validity, reinforcing the need for cautious interpretation. To address this, our modelling framework included a sensitivity analysis in which clinically suspected cases were reclassified as non-cases (sensitivity analysis 4), and the same predictors emerged as stable. Six risk factors in adolescence were associated with endometriosis. Three factors relate to severity of general pelvic pain, two to menstrual disorders and one with a family history of the condition. The resultant SAFE score was calculated as the sum of the individual factors to produce a risk summary. However, further evaluation with clinicians and patients is still needed to establish its performance in practice.

## Contributors

GDM, GWM, JD, AJD, and JA conceptualised the project. GDM, MRB, and AJD worked on methodology, and accessed and verified the data. MRB analysed the data. GDM, MRB, AJD, and SM wrote the manuscript. GDM, SM, GWM, JD, and JA reviewed the manuscript. All authors read and approved the final version of the manuscript.

## Data sharing statement

Researchers should contact the principal investigator (GDM) to use GELLES survey data. Access to linked health data requires approval from Human Research Ethics Committees and Data Custodians.

## Declaration of interests

JA is on advisory boards and received consulting fees from Gedeon Richter, BD, Bayer and Hologic, received honoraria from Hologic, and received support for attending meetings from Gedeon Richter. He is chair of the Australian Endometriosis Guideline Committee, past-President of AGES, Co-Editor in Chief of JMIG and holds multiple competitive grants in endometriosis research through Australian Government funding agencies (Commonwealth Department of Health and Aged Care, MRFF), AGES and Country Women's Association of NSW. GDM, MRB, SM, GWM, JD and AJD declare that they have no known competing financial interests or personal relationships that could have appeared to influence the work reported in this paper. The authors declare that they have no known competing financial interests or personal relationships that could have appeared to influence the work reported in this paper.

## References

[1] Giudice L.C., Kao L.C. (2004). Endometriosis. Lancet.

[2] Rowlands I.J., Abbott J.A., Montgomery G.W., Hockey R., Rogers P., Mishra G.D. (2021). Prevalence and incidence of endometriosis in Australian women: a data linkage cohort study. BJOG.

[3] Soliman A.M., Yang H., Du E.X., Kelley C., Winkel C. (2016). The direct and indirect costs associated with endometriosis: a systematic literature review. Hum Reprod.

[4] Armour M., Lawson K., Wood A., Smith C.A., Abbott J. (2019). The cost of illness and economic burden of endometriosis and chronic pelvic pain in Australia: a national online survey. PLoS One.

[5] O'Hara R., Rowe H., Fisher J. (2022). Managing endometriosis: a cross-sectional survey of women in Australia. J Psychosom Obstet Gynecol.

[6] Armour M., Sinclair J., Ng C.H.M. (2020). Endometriosis and chronic pelvic pain have similar impact on women, but time to diagnosis is decreasing: an Australian survey. Sci Rep.

[7] Hudelist G., Fritzer N., Thomas A. (2012). Diagnostic delay for endometriosis in Austria and Germany: causes and possible consequences. Hum Reprod.

[8] Zondervan K.T., Becker C.M., Missmer S.A. (2020). Endometriosis. N Engl J Med.

[9] Crump J., Suker A., White L. (2024). Endometriosis: a review of recent evidence and guidelines. Aust J Gen Pract.

[10] Moss K.M., Doust J., Homer H., Rowlands I.J., Hockey R., Mishra G.D. (2021). Delayed diagnosis of endometriosis disadvantages women in ART: a retrospective population linked data study. Hum Reprod.

[11] Surrey E., Soliman A.M., Trenz H., Blauer-Peterson C., Sluis A. (2020). Impact of endometriosis diagnostic delays on healthcare resource utilization and costs. Adv Ther.

[12] Horne A.W., Saunders P.T.K., Abokhrais I.M., Hogg L. (2017). Endometriosis Priority Setting Partnership Steering Group (appendix). Top ten endometriosis research priorities in the UK and Ireland. Lancet.

[13] Armour M., Ciccia D., Yazdani A. (2023). Endometriosis research priorities in Australia. Aust N Z J Obstet Gynaecol.

[14] (2021). Royal Australian and New Zealand College of Obstetricians and Gynaecologists Australian clinical practice guideline for the diagnosis and management of endometriosis.

[15] National Institute for Health and Care Excellence (NICE) (2017). Endometriosis: diagnosis and management (NICE guideline NG73). http://www.nice.org.uk/guidance/ng73.

[16] Becker C.M., Bokor A., Heikinheimo O. (2022). ESHRE guideline: endometriosis. Hum Reprod Open.

[17] Rajesh S., Mehmeti A., Smith-Walker T., Kendall B. (2025). Diagnosis and management of endometriosis: summary of updated NICE guidance. BMJ.

[18] Surrey E., Carter C.M., Soliman A.M., Khan S., DiBenedetti D.B., Snabes M.C. (2017). Patient-completed or symptom-based screening tools for endometriosis: a scoping review. Arch Gynecol Obstet.

[19] Gkrozou F., Tsonis O., Sorrentino F. (2024). Endometriosis predictive models based on self-assessment questionnaire, evidence from clinical examination or imaging findings: a narrative review. J Clin Med.

[20] Goldstein A., Cohen S. (2023). Self-report symptom-based endometriosis prediction using machine learning. Sci Rep.

[21] Fauconnier A., Drioueche H., Huchon C. (2021). Early identification of women with endometriosis by means of a simple patient-completed questionnaire screening tool: a diagnostic study. Fertil Steril.

[22] Lee C., Dobson A.J., Brown W.J. (2005). Cohort profile: the Australian longitudinal study on women's health. Int J Epidemiol.

[23] Loxton D., Tooth L., Harris M.L. (2018). Cohort profile: the Australian longitudinal study on women's health (ALSWH) 1989–95 cohort. Int J Epidemiol.

[24] Austin P.C., Tu J.V. (2004). Automated variable selection methods for logistic regression produced unstable models for predicting acute myocardial infarction mortality. J Clin Epidemiol.

[25] Austin P.C., Tu J.V. (2004). Bootstrap methods for developing predictive models. Am Statistician.

[26] Sauerbrei W., Schumacher M. (1992). A bootstrap resampling procedure for model building: application to the Cox regression model. Stat Med.

[27] Nisenblat V., Bossuyt P.M., Shaikh R. (2016). Blood biomarkers for the non-invasive diagnosis of endometriosis. Cochrane Database Syst Rev.

[28] Verket N.J., Falk R.S., Qvigstad E., Tanbo T.G., Sandvik L. (2019). Development of a prediction model to aid primary care physicians in early identification of women at high risk of developing endometriosis: cross-sectional study. BMJ Open.

[29] Khashchenko E.P., Uvarova E.V., Fatkhudinov T.K. (2023). Endometriosis in adolescents: diagnostics, clinical and laparoscopic features. J Clin Med.

[30] Treloar S.A., Bell T.A., Nagle C.M., Purdie D.M., Green A.C. (2010). Early menstrual characteristics associated with subsequent diagnosis of endometriosis. Am J Obstet Gynecol.

[31] Gete D.G., Dobson A.J., Mortlock S. (2025). Validation of self-reported endometriosis. Maturitas.

