# Supplementary material for: Development of an early prediction model for endometriosis risk: the simplified adolescent factors for endometriosis (SAFE) score
Source: eClinicalMedicine. 2026 Mar 9;93:103806. doi: 10.1016/j.eclinm.2026.103806 (PMC13043476; doi:10.1016/j.eclinm.2026.103806)
Supplement: Supplementary Materials [file mmc1.docx]

**Supplementary Text S1: Survey Questions**

Questions related to pelvic pain in general (not related to periods) included frequency of occurrence (never/rarely/sometimes versus often/very often); usual length of an episode (< one hour versus ≥ one hour); sought treatment for pain (no versus yes); severity of pelvic pain; pelvic pain made it difficult to participate in daily work or school activities; pelvic pain made it difficult to participate in daily social or recreational activities. Responses related to the severity of pelvic pain, difficulties in participating in daily work or school activities, and social or recreational activities were measured on a scale of 0 to 10 and categorised into three groups: 1 to 3, 4 to 6, and 7 to 10, where higher values indicated more severe pain or greater impact on daily activities.

Menstrual-related characteristics assessed included menstrual patterns (regular, irregular, no pattern, or on contraception), cycle length (≥ 24 days, < 24 days, too irregular, on contraception), menstrual flow (spotting/light/moderate, heavy, on contraception), and age at menarche (≤ 11 versus > 11 years). Moreover, women were asked “Has there been a time in your life when you typically had pelvic pain during your periods?” Those who responded yes were classified as having painful periods. They were then asked to report the age at which the pain began.

Information regarding general pelvic pain and its consequences and menstrual-related risk factors was collected for different stages of life: up to the age of 15 years, from age 16 to 19 years, 20 to 29, and after 30 years. In the 1973-78 cohort, similar information was collected for the stages up to the age of 15 years, from 16 to 19, 20 to 29, 30 to 39, and after age 40 years.

Exposure to tobacco smoke in utero and in early life was obtained from the questions on whether the woman’s biological mother smoked cigarettes or any tobacco products while she was pregnant with the index woman (yes, no, don’t know); and whether their biological mother, and or biological father smoked cigarettes or any tobacco products on a daily basis in the home when the index woman was a child (yes, no, don’t know).

Family history of endometriosis was ascertained from the questions “has your biological mother, sisters, grandmother, biological aunts or biological cousins on your mother’s side and “has your grandmother, biological aunts or cousin on your father’s side been diagnosed or treated from endometriosis”. The response options were yes, no, don’t know or not applicable (for biological sisters, aunts, cousins). Women were classified as having a family history of endometriosis if they answered ‘yes’ for any family member.

**Supplementary Table S1:** Diagnostic performance (measured by AUC) of risk factors related to pelvic pain and menstrual characteristics across different stages of life in the derivation sample (1989-95 sample)

| Life stage | Up to the age of 15 | Between the ages of 16 to 19 |
| --- | --- | --- |
| **Risk factors related to pelvic pain and its consequences** | | |
| How often did you have pelvic pain? (do not count pain related to period, intercourse, pregnancy or childbirth, surgery, injury, food poisoning, or stomach flu) | 0.57 | 0.62 |
| How long did an episode of pelvic pain usually last? | 0.59 | 0.62 |
| Did you seek treatment for this pain? | 0.59 | 0.64 |
| When you had this pelvic pain, how difficult did the pain make it for you to participate in daily work or school activities? | 0.62 | 0.66 |
| When you had this pelvic pain, how difficult did the pain make it for you to participate in daily social or recreational activities? | 0.62 | 0.66 |
| Did you ever take painkillers for this pain? | 0.63 | 0.66 |
| Thinking about your pelvic pain, please rate how severe your pelvic pain was | 0.64 | 0.67 |
| **Menstrual risk factors** | | |
| Menstrual regularity | 0.57 | 0.60 |
| Cycle length | 0.56 | 0.59 |
| Menstrual flow | 0.58 | 0.61 |

**Supplementary Table S2:** Distribution of experience and severity of pelvic pain and its consequences between ages 16 to 19 in the 1973-78 and 1989-95 cohorts

| Risk factor | Category | 1973-78 cohort | | | 1989-95 cohort | | |
| --- | --- | --- | --- | --- | --- | --- | --- |
|  |  | without endometriosis (n=3,419) | Confirmed endometriosis (n=514) | Suspected endometriosis  (n=144) | without endometriosis (n=4,755) | Confirmed endometriosis (n=406) | Suspected endometriosis  (n=179) |
| How often did you have pelvic pain? (do not count pain related to period, intercourse, pregnancy or childbirth, surgery, injury, food poisoning, or stomach flue) | Never/ rare/ sometimes | 3,064 (89.6%) | 398 (77.4%) | 99 (68.8%) | 4,166 (87.6%) | 250 (61.6%) | 130 (72.6%) |
|  | Often/ very often | 134 (3.9%) | 93 (18.1%) | 15 (10.4%) | 227 (4.8%) | 140 (34.5%) | 24 (13.4%) |
|  | Missing | 221 (6.5%) | 23 (4.5%) | 30 (20.8%) | 362 (7.6%) | 16 (3.9%) | 25 (14.0%) |
| How long an episode of pelvic pain last | Up to 1 hour | 2,961 (86.6%) | 382 (74.3%) | 93 (64.6%) | 4,013 (84.4%) | 239 (58.9%) | 115 (64.2%) |
|  | More than 1 hours | 232 (6.8%) | 108 (21.0%) | 21 (14.6%) | 372 (7.8%) | 151 (37.2%) | 38 (21.2%) |
|  | Missing | 226 (6.6%) | 24 (4.7%) | 30 (20.8%) | 370 (7.8%) | 16 (3.9%) | 26 (14.5%) |
| Did you seek treatment for this pain? | No | 3,073 (89.9%) | 393 (76.5%) | 93 (64.6%) | 4,086 (85.9%) | 221 (54.4%) | 121 (67.6%) |
|  | Yes | 121 (3.5%) | 98 (19.1%) | 21 (14.6%) | 298 (6.3%) | 167 (41.1%) | 33 (18.4%) |
|  | Missing | 225 (6.6%) | 23 (4.5%) | 30 (20.8%) | 371 (7.8%) | 18 (4.4%) | 25 (14.0%) |
| When you had this pelvic pain, how difficult did the pain make it for you to participate in daily work or school activities? | 0 to 3 | 2,914 (85.2%) | 354 (68.9%) | 89 (61.8%) | 3,783 (79.6%) | 183 (45.1%) | 99 (55.3%) |
|  | 4 to 6 | 165 (4.8%) | 51 (9.9%) | 10 (6.9%) | 368 (7.7%) | 78 (19.2%) | 30 (16.8%) |
|  | 7 to 10 | 104 (3.3%) | 81 (15.8%) | 15 (10.4%) | 237 (5.0%) | 128 (31.5%) | 25 (14.0%) |
|  | Missing | 236 (6.9%) | 28 (5.4%) | 30 (20.8%) | 367 (7.7%) | 17 (4.2%) | 25 (14.0%) |
| When you had this pelvic pain, how difficult did the pain make it for you to participate in daily social or recreational activities? | 0 to 3 | 2,905 (85.0%) | 349 (67.9%) | 89 (61.8%) | 3,810 (80.1%) | 185 (45.6%) | 102 (57.0%) |
|  | 4 to 6 | 153 (4.5%) | 51 (9.9%) | 8 (5.6%) | 339 (7.1%) | 79 (19.5%) | 27 (15.1%) |
|  | 7 to 10 | 106 (3.1%) | 83 (16.1%) | 14 (9.7%) | 238 (5.0%) | 125 (30.8%) | 25 (14.0%) |
|  | Missing | 255 (7.5%) | 31 (6.0%) | 33 (22.9%) | 368 (7.7%) | 17 (4.2%) | 25 (14.0%) |
| Did you ever take painkillers for this pain? | No | 2,766 (80.9%) | 328(63.8%) | 83 (57.6%) | 3,537 (74.4%) | 158 (38.9%) | 87 (48.6%) |
|  | Yes | 426 (12.5%) | 163 (31.7%) | 30 (20.8%) | 846 (17.8%) | 229 (56.4%) | 67 (37.4%) |
|  | Missing | 227 (6.6%) | 23 (4.5%) | 31 (21.5%) | 372 (7.8%) | 19 (4.7%) | 25 (14.0%) |
| Thinking about your pelvic pain, please rate how severe your pelvic pain was | 0 to 3 | 2,812 (82.2%) | 332 (64.6%) | 87 (60.4%) | 3,580 (75.3%) | 164 (40.4%) | 89 (49.7%) |
|  | 4 to 6 | 206 (6.0%) | 51 (9.9%) | 9 (6.2%) | 434 (9.1%) | 59 (14.5%) | 29 (16.2%) |
|  | 7 to 10 | 166 (4.9%) | 103 (20.0%) | 17 (11.8%) | 372 (7.8%) | 166 (40.9%) | 36 (20.1%) |
|  | Missing | 235 (6.9%) | 28 (5.4%) | 31 (21.5%) | 369 (7.8%) | 17 (4.2%) | 25 (14.0%) |

Column percentages are reported.

**Supplementary Table S3:** Distribution of cycle length, period irregularity, and menstrual flow between ages 16 to 19; experience of painful period up to the age of 19; and age at menarche in the 1973-78 and 1989-95 cohorts

| Risk factor | Category | 1973-78 cohort | | | 1989-95 cohort | | |
| --- | --- | --- | --- | --- | --- | --- | --- |
|  |  | without endometriosis (n=3,419) | Confirmed endometriosis (n=514) | Suspected endometriosis  (n=144) | without endometriosis (n=4,755) | Confirmed endometriosis (n=406) | Suspected endometriosis  (n=179) |
| Period regularity | Regular | 2,113 (61.8%) | 264 (51.4%) | 74 (51.4%) | 1,951 (41.0%) | 87 (21.4%) | 49 (27.4%) |
|  | Irregular | 310 (9.1%) | 62 (12.1%) | 14 (9.7%) | 593 (12.5%) | 49 (12.1%) | 32 (17.9%) |
|  | No pattern | 110 (3.2%) | 17 (3.3%) | 5 (3.5%) | 152 (3.2%) | 24 (5.9%) | 9 (5.0%) |
|  | On contraception | 753 (22.0%) | 154 (30.0%) | 31 (21.5%) | 1,822 (38.3%) | 233 (57.4%) | 71 (39.7%) |
|  | Missing | 133 (3.9%) | 17 (3.3%) | 20 (13.9%) | 237 (5.0%) | 13 (3.2%) | 18 (10.1%) |
| Cycle length | <24 days | 212 (6.2%) | 57 (11.1%) | 9 (6.2%) | 121 (2.5%) | 18 (4.4%) | 7 (3.9%) |
|  | ≥ 24 days | 2,147 (62.8%) | 266 (51.8%) | 76 (52.8%) | 2,273 (47.8%) | 107 (26.4%) | 67 (37.4%) |
|  | Too irregular | 163 (4.8%) | 23 (4.5%) | 7 (4.9%) | 297 (6.2%) | 34 (8.4%) | 16 (8.9%) |
|  | On contraception | 753 (22.0%) | 154 (30.0%) | 31 (21.5%) | 1,822 (38.3%) | 233 (57.4%) | 71 (39.7%) |
|  | Missing | 144 (4.2%) | 14 (2.7%) | 21 (14.6%) | 242 (5.1%) | 14 (3.4%) | 18 (10.1%) |
| Menstrual flow on average | Spotting/ light/ moderate | 2,149 (62.9%) | 215 (41.8%) | 64 (44.4%) | 2,357 (49.6%) | 99 (24.4%) | 75 (41.9%) |
|  | Heavy | 368 (10.8%) | 130 (25.3%) | 30 (20.8%) | 322 (6.8%) | 61 (15.0%) | 16 (8.9%) |
|  | On contraception | 753 (22.0%) | 154 (30.0%) | 31 (21.5%) | 1,822 (38.3%) | 233 (57.4%) | 71 (39.7%) |
|  | Missing | 149 (4.4%) | 15 (2.9%) | 19 (13.2%) | 254 (5.3%) | 13 (3.2%) | 17 (9.5%) |
| Painful period up to the age of 19 | No | 2602 (76.1%) | 212 (41.2%) | 57 (39.6%) | 3349 (70.4%) | 107 (26.4%) | 56 (31.3%) |
|  | Yes | 571 (16.7%) | 263 (51.2%) | 57 (39.6%) | 994 (20.9%) | 266 (65.5%) | 102 (57.0%) |
|  | Missing | 246 (7.2%) | 39 (7.6%) | 30 (20.8%) | 412 (8.7%) | 33 (8.1%) | 21 (11.7%) |
| Age at menarche | ≤ 11 years | 381 (11.1%) | 77 (15.0%) | 20 (13.9%) | 833 (17.5%) | 86 (21.2%) | 39 (21.8%) |
|  | > 11 years | 2,537 (74.2%) | 366 (71.2%) | 103 (71.5%) | 3,887 (81.7%) | 319 (78.6%) | 139 (77.7%) |
|  | Missing | 501 (14.7%) | 71 (13.8%) | 21 (14.6%) | 35 (0.7%) | 1 (0.2%) | 1 (0.6%) |

Column percentages are reported.

**Supplementary Table S4:** Distribution of exposure to smoking during childhood in the 1973-78 and 1989-95 cohorts

| Risk factor | Category | 1973-78 cohort | | | 1989-95 cohort | | |
| --- | --- | --- | --- | --- | --- | --- | --- |
|  |  | without endometriosis (n=3,419) | Confirmed endometriosis (n=514) | Suspected endometriosis  (n=144) | without endometriosis (n=4,755) | Confirmed endometriosis (n=406) | Suspected endometriosis  (n=179) |
| Mother smoking during pregnancy | No | 2,351(68.8%) | 339 (66.0%) | 80 (55.6%) | 3,528 (74.2%) | 299 (73.6%) | 121 (67.6%) |
|  | Yes | 448 (13.1%) | 78 (15.2%) | 21 (14.6%) | 503 (10.6%) | 62 (15.3%) | 28 (15.6%) |
|  | Don’t now | 278 (8.1%) | 51 (9.9%) | 6 (4.2%) | 382 (8.0%) | 32 (7.9%) | 10 (5.6%) |
|  | Missing | 342 (10.0%) | 46 (8.9%) | 37 (25.7%) | 342 (7.2%) | 13 (3.2%) | 20 (11.2%) |
| Mother smoking during childhood | No | 2,417 (70.7%) | 352 (68.5%) | 80 (55.6%) | 3,726 (78.4%) | 311 (76.6%) | 124 (69.3%) |
|  | Yes | 643 (18.8%) | 114 (22.2%) | 25 (17.4%) | 597 (12.6%) | 76 (18.7%) | 30 (16.8%) |
|  | Missing | 359 (10.5%) | 48 (9.3%) | 39 (27.1%) | 432 (9.1%) | 19 (4.7%) | 25 (14.0%) |
| Father smoking during childhood | No | 2,145 (62.7%) | 338 (65.8%) | 69 (47.9%) | 3,758 (79.0%) | 316 (77.8%) | 125 (69.8%) |
|  | Yes | 915 (26.8%) | 128 (24.9%) | 36 (25.0%) | 565 (11.9%) | 71 (17.5%) | 29 (16.2%) |
|  | Missing | 352 (10.5%) | 48 (9.3%) | 39 (27.1%) | 432 (9.1%) | 19 (4.7%) | 25 (14.0%) |

Column percentages are reported.

**Supplementary Table S5:** Distribution of preterm birth and family history of endometriosis in the 1973-78 and 1989-95 cohorts

| Risk factor | Category | 1973-78 cohort | | | 1989-95 cohort | | |
| --- | --- | --- | --- | --- | --- | --- | --- |
|  |  | without endometriosis (n=3,419) | Confirmed endometriosis (n=514) | Suspected endometriosis  (n=144) | without endometriosis (n=4,755) | Confirmed endometriosis (n=406) | Suspected endometriosis  (n=179) |
| having been born preterm | No | 2,742 (80.2%) | 417 (81.1%) | 95 (66.0%) | 3,876 (81.5%) | 342 (84.2%) | 140 (78.2%) |
|  | Yes | 200 (5.8%) | 36 (7.0%) | 5 (3.5%) | 416 (8.7%) | 37 (9.1%) | 14 (7.8%) |
|  | Don’t know | 135 (3.9%) | 19 (3.7%) | 5 (3.5%) | 132 (2.8%) | 14 (3.4%) | 4 (2.2%) |
|  | Missing | 342 (10.0%) | 42 (8.2%) | 39 (27.1%) | 331 (7.0%) | 13 (3.2%) | 21 (11.7%) |
| Family history of endometriosis | Yes | 519 (15.2%) | 164 (31.9%) | 31 (21.5%) | 778 (16.4%) | 195 (48.0%) | 71 (39.7%) |
|  | No (including don’t know/ not applicable/ missing) | 2900 (84.8%) | 350 (68.1%) | 113 (78.5%) | 3977 (83.6%) | 211 (52.0%) | 108 (60.3%) |

Column percentages are reported.

**Supplementary Text S2: Calibration of the model developed using training sample**

To assess the calibration of the model, the regression coefficients estimated using training data (i.e., 75% of the 1989-95 cohort) were multiplied by the variables to predict the probability of having endometriosis. We divided the estimated probabilities of endometriosis into ten deciles. In each decile, the average of estimated probabilities and the observed proportion of women who reported endometriosis was calculated. The agreement between observed and predicted probabilities was assessed by the Bland-Altman plot and the Hosmer-Lemeshow test of calibration (1).

Figure S1 top panel shows the observed versus predicted probabilities in the training sample. The Hosmer-Lemeshow goodness of fit test had a p-value of 0.56, indicating calibration of the model. Figure 1 bottom panel shows the Bland-Altman plot illustrating the difference between observed and predicted probabilities in the training sample. No systematic bias in the estimation of the risk of endometriosis was observed. The differences fell within the limits of agreement suggesting good agreement between observed and predicted probabilities.

**Supplementary Figure S1:** Assessment of agreement between observed and predicted probabilities in training sample: calibration (top panel) and Bland-Altman plot (bottom panel)


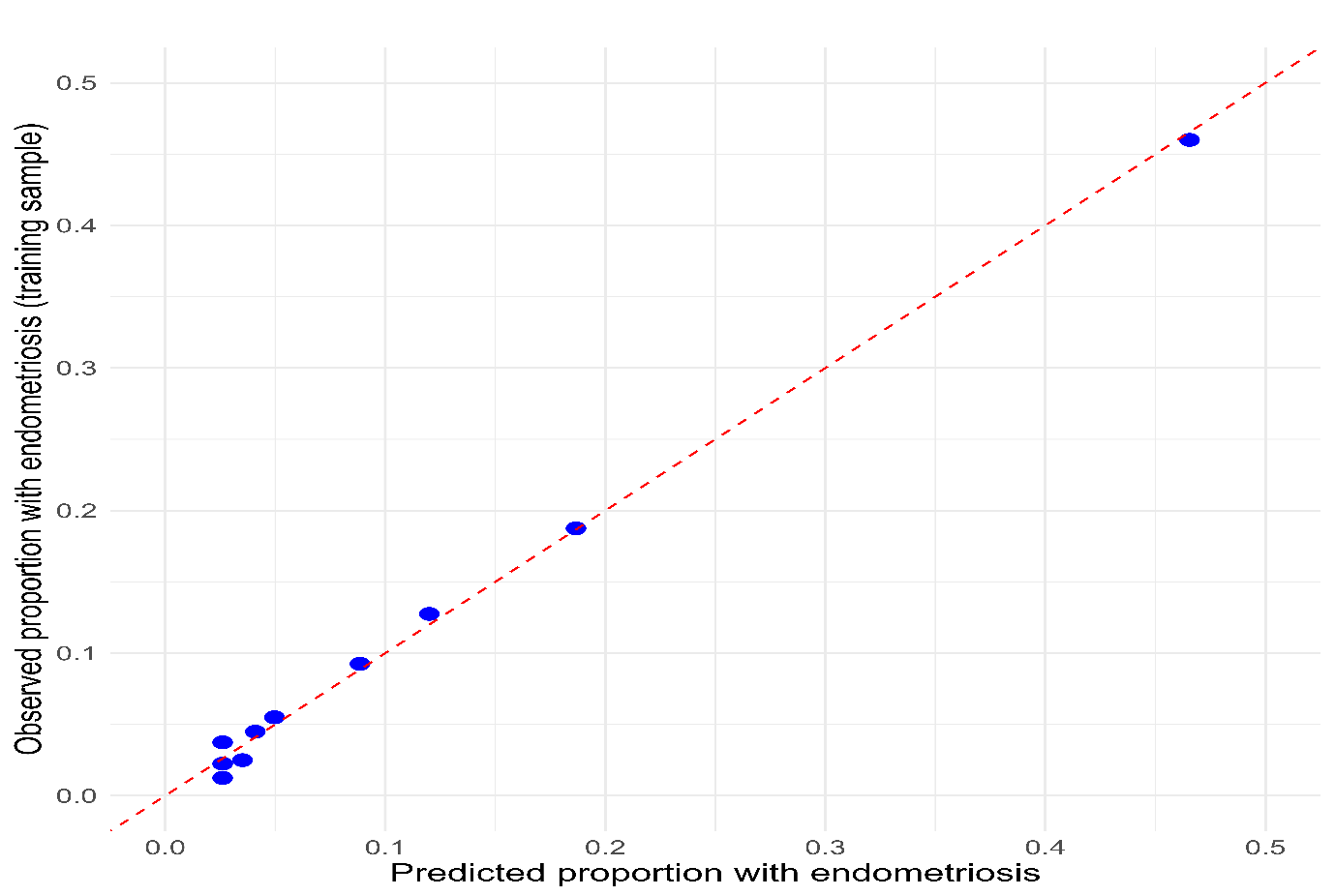

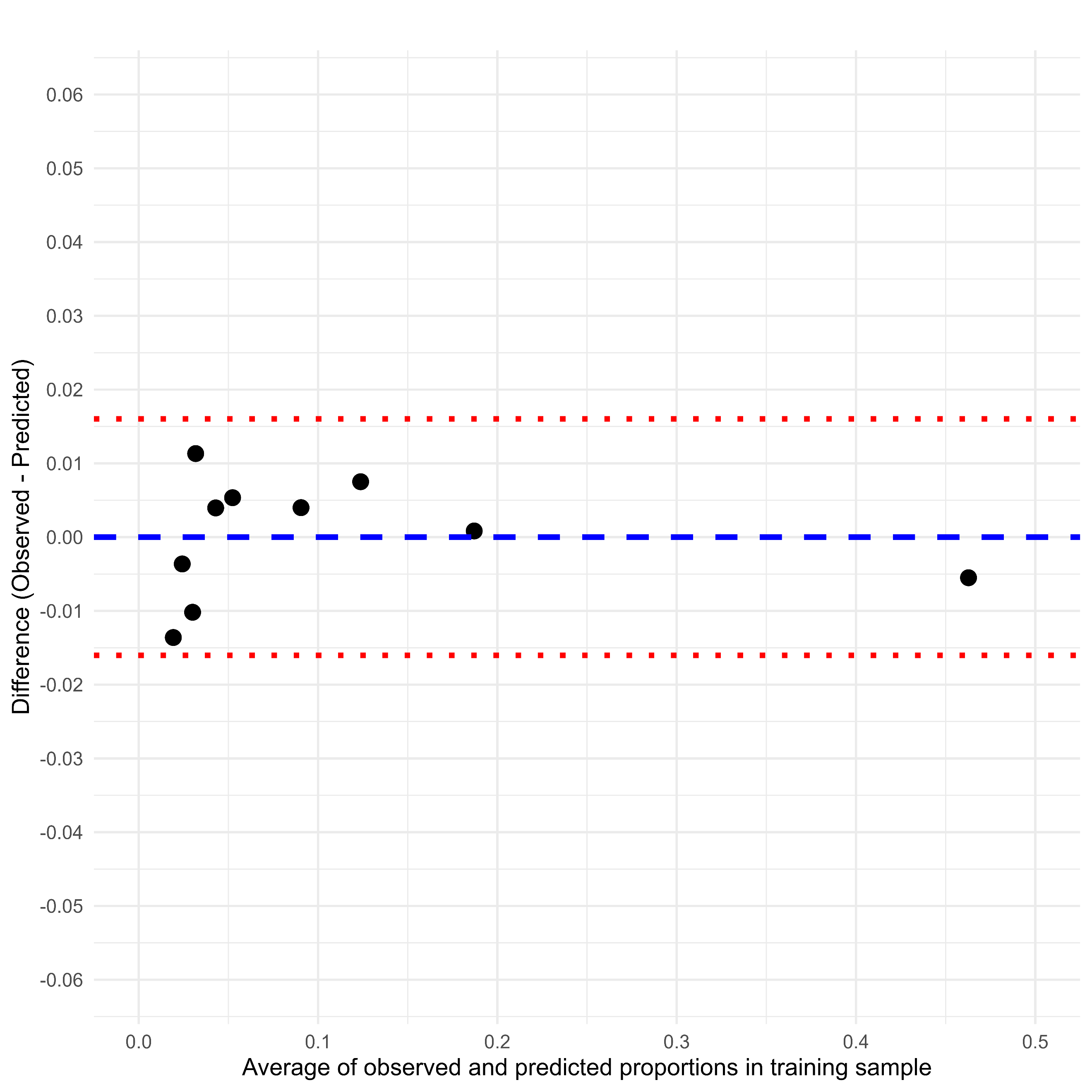


**Supplementary Table S6:** Association between stable risk factors and endometriosis in the external sample of the 1973-78 cohort

| Risk factor | OR (95% CI) |
| --- | --- |
| How often did you have pelvic pain? (do not count pain related to period, intercourse, pregnancy or childbirth, surgery, injury, food poisoning, or stomach flu)   - Never/ rare/ sometimes - Often/ very often | 1.00  1.56 (1.10, 2.22) |
| Did you seek treatment for this pain?   - No - Yes | 1.00  2.83 (2.00, 3.99) |
| Menstrual flow   - Spotting/ light/ moderate - Heavy - On contraception | 1.00  1.91 (1.50, 2.43) 1.38 (1.11, 1.71) |
| Painful periods up to the age of 19   - No - Yes | 1.00  3.20 (2.64, 3.88) |
| History of endometriosis in other family members   - No (including don’t know) - Yes | 1.00  1.82 (1.48, 2.24) |

Stable risk factors were defined as those that had a p-value < 0.05 in more than 50% of bootstrap samples.

**Supplementary Table S7:** Association between the most recent values of stable risk factors and endometriosis in the training sample (75% of the derivation sample of data from the 1989-95 cohort)

| Risk factor | OR (95% CI) |
| --- | --- |
| How often did you have pelvic pain? (do not count pain related to period, intercourse, pregnancy or childbirth, surgery, injury, food poisoning, or stomach flu)   - Never/ rare/ sometimes - Often/ very often | 1.00  1.88 (1.32, 2.68) |
| Did you seek treatment for this pain?   - No - Yes | 1.00  1.42 (1.02, 2.01) |
| Did you ever take painkillers for this pain?   - No - Yes | 1.00  1.50 (1.11, 2.01) |
| Menstrual flow   - Spotting/ light/ moderate - Heavy - On contraception | 1.00  1.27 (0.88, 1.82) 1.18 (1.02, 1.44) |
| Painful periods up to the age of 19   - No - Yes | 1.00  4.68 (3.72, 5.89) |
| History of endometriosis in other family members   - No (including don’t know) - Yes | 1.00  3.43 (2.72, 4.31) |

Stable risk factors were defined as those that had a p-value < 0.05 in more than 50% of bootstrap samples.

For example, for a woman diagnosed with endometriosis at the age of 35, recent risk factors refer to the risk factors at ages 20 to 29 years.

**Supplementary Table S8:** Assessment of internal and external validity of the model ^a^ using the sum of the number of risk factors when women with suspected endometriosis were treated not having endometriosis

| Statistics | Cut-off point | Training sample (75% of 1989-95 cohort; N=4,005) | Internal test sample (25% of 1989-95 cohort; N=1,335) | External test sample  (1973-78 cohort; N=4,077) |
| --- | --- | --- | --- | --- |
| Proportion of women with endometriosis |  | 10.6 % | 11.9 % | 16.1 % |
| Sensitivity (%) | 1 | 90.3 | 88.0 | 77.6 |
|  | 2 | 65.7 | 68.4 | 50.9 |
|  | 3 | 47.1 | 51.2 | 26.3 |
| Specificity (%) | 1 | 54.3 | 53.4 | 59.1 |
|  | 2 | 82.2 | 81.4 | 85.1 |
|  | 3 | 92.2 | 90.8 | 94.0 |
| PPV (%) | 1 | 13.3 | 15.4 | 21.3 |
|  | 2 | 22.4 | 26.1 | 32.9 |
|  | 3 | 32.0 | 65.1 | 38.9 |
| NPV (%) | 1 | 98.6 | 97.9 | 94.6 |
|  | 2 | 96.8 | 96.4 | 92.3 |
|  | 3 | 95.7 | 95.1 | 90.0 |
| AUC |  | 0.82 (0.79, 0.84) | 0.81 (0.77, 0.85) | 0.73 (0.70, 0.75) |

a: The model was developed using training data and its performance was assessed on training, internal test, and external samples.

In the main analysis, women with suspected endometriosis were considered as having endometriosis and performance statistics were calculated. As a sensitivity analysis, women with suspected endometriosis were treated as not having endometriosis.

**Supplementary Text S3: Presenting the model and prediction**

To estimate the probability of having endometriosis for an individual woman, the regression coefficients derived from the training data (i.e., 75% of the 1989-95 cohort) are multiplied by the corresponding variables (Formula S1). The estimated coefficients are summarised in Supplementary Table S9. In Formula 1, $X_{1}$ to $X_{k}$ represent risk factors and $\beta_{1}$ to $\beta_{k}$ are the estimated regression coefficients. The parameter $\beta_{0}$ denotes the intercept of the model, which reflects the baseline risk.

**Supplementary Table S9:** Estimated regression coefficients in the training sample (75% of 1989-95 cohort; N=4,005)

| Variable | Coefficient (β) |
| --- | --- |
| Intercept | -3.53 |
| Menstrual flow: heavy | 0.23 |
| Menstrual flow: on contraception | 0.46 |
| Family history of endometriosis: positive | 1.19 |
| Painful period: yes | 1.42 |
| Pelvic pain in general: Often | 0.57 |
| Seeking treatment for pelvic pain: yes | 0.75 |
| Taking painkillers for pelvic pain: yes | 0.51 |

$$Formula S1: Probability of endometriosis=\frac{exp(\beta_{0}+\beta_{1}X_{1}+\ldots+\beta_{k}X_{k})}{1+exp(\beta_{0}+\beta_{1}X_{1}+\ldots+\beta_{k}X_{k})}$$

1. LEMESHOW S, HOSMER DW, JR. A REVIEW OF GOODNESS OF FIT STATISTICS FOR USE IN THE DEVELOPMENT OF LOGISTIC REGRESSION MODELS1. American Journal of Epidemiology. 1982;115(1):92-106.
